# Milk recording data indicates the importance of fertility, including age at first calving, on the progression of first lactation cows to second lactation

**DOI:** 10.1371/journal.pone.0297657

**Published:** 2024-01-29

**Authors:** Emma N. Taylor, Kulwant Channa, James Hanks, Nick M. Taylor

**Affiliations:** 1 PAN Livestock Services Ltd., School of Agriculture, Policy and Development, University of Reading, Reading, United Kingdom; 2 Veterinary Epidemiology and Economics Research Unit (VEERU), School of Agriculture, Policy and Development, University of Reading, Reading, United Kingdom; Damanhour University, EGYPT

## Abstract

Dairy farmers do not recoup the rearing costs incurred from birth to first calving until second lactation but varying proportions of first lactation cows are removed from the herd before second calving. Herein, we used milk recording data to examine the outcomes and performance of first lactation cows to gain insight into farmer decisions to keep or remove them from the herd. An InterHerd+ dataset derived from 500 milk recording dairy herds in UK was used to examine first lactation cows which calved in 2020. Of 29,128 first lactation cows that calved in 2020, 82.6% remained within the herd and re-calved, 4.9% conceived but exited the herd before re-calving, 6.0% were served but exited the herd after failing to conceive and 6.6% exited the herd without being served. The fertility data on these cows support the logical conclusion that farmers retain cows that are served and conceive sooner, possibly in order to keep within a broadly seasonal calving pattern. Cows which were served but not conceived had a median AFC 16–20 days greater than the median AFC for those that conceived. Farmers may also be retaining cows with relatively high milk yields and lower somatic cell counts, or these parameters may be an indicator of a range of attributes affecting the farmer’s decision. The data also suggest that farmers are rearing more replacements than required, because over one third of the cows removed in first lactation are never served, and 70% of these are sold within 120 days post-partum. These cows had a significantly older median age at first calving of 818 days, but their early removal without serving suggests there is an oversupply of replacements forcing farmers to dispose of these cows early in lactation. In order to develop a deeper understanding of herd turnover and replacement, future work could examine cow removals in lactation 2 onwards.

## Introduction

UK dairy heifers incur mean (± standard deviation) total birth to first calving costs of £1819 ± 387/heifer and farmers do not recoup these costs from milk production income until the cow is in its second lactation, 530 days after first calving [1]. Despite the high initial investment, a proportion of first lactation cows are not retained within the herd into the second lactation. UK-based literature relating specifically to the culling of first lactation cows is limited and is over 10 years old. For example, an examination of data from 18 dairy farms containing 468 Holstein Friesian (HF) first lactation cows observed that of those that calved, 19% were culled without progressing to second lactation. Of the first lactation cows which were culled, 37% were culled due to infertility [2]. Published literature from other countries gives a varied picture. In the USA, De Vries [3] reported that 29% of first lactation Holstein cows did not progress to second lactation. A study in Sweden reported that 28% of conventionally managed and 25% of organic first lactation Holstein cows did not progress to second lactation [4]. In the Swedish study, poor fertility (conventional, 32% of culls; organic, 31% of culls), udder health (conventional, 15%; organic, 21%) and low milk production (9% of culls in both types) were the most common reasons given for first lactation cows not progressing to second lactation [4]. Likewise, in Poland, first lactation HF cows are frequently culled due to poor fertility (43.9%) and udder diseases (13.0%) [5]. In Estonia, 16% of first lactation Holstein, Red and Native cows did not progress to second lactation, of which 25% were culled due to feet/claw disorders, 18% due to udder disorders and 15% due to fertility problems [6].

Milk recording data enables farmers to make more informed management decisions by providing individual cow data on a range of parameters, including; milk yield and composition, fertility and health. Milk recording herds are more economically sustainable due to increased gross margins and milk yields per cow [7]. However, the use of milk recording varies: in 2015, 39%, 69% and 68% of dairy herds milk recorded across Ireland, France and Germany, respectively [8]. Herds with a larger herd size are more likely to milk record. Thus, the proportion of dairy cows with milk recording data is larger than the proportion of herds with milk recording data. In 2015, 52%, 69% and 88% of dairy cows were milk recorded across Ireland, France and Germany, respectively [8]. Data from the Agriculture and Horticulture Development Board (AHDB) [9] on the number of dairy producers in Great Britain and results from a milk recording systems survey [8] suggest that approximately 50–55% of dairy herds in England and Wales collect milk recording data. Data from 500 milk recording herds has been used to monitor UK dairy production parameters including annual cull rates since 2010 [10]. However, the proportion of first lactation cows which progress to second lactation within the same herd and influential factors affecting this progression have been not determined. Identifying and understanding the factors influencing UK farmers’ decisions to remove first lactation cows from the herd could lead to finding techniques to mitigate these factors. Improving the efficiency of dairy replacement supply, combined with extending the productive lifespan of dairy cows to reduce overall replacement requirement could reduce the environmental impact of milk production and alleviate public concerns regarding dairy cattle welfare [11].

Herein, we use milk recording data from 500 randomly sampled milk recording herds to examine the outcomes and performance of first lactation cows and determine the proportion of first lactation cows which progress to second lactation in the same herd. Associated milk recording data is also used to gain insight into factors that may influence farmer decisions to keep or remove them from the herd.

## Materials and methods

### Data source

This study made use of the large InterHerd+ dataset used for the annual 500-herd Key Performance Indicator (KPI) reports produced by the University of Reading (PAN Livestock Services Ltd., 2023). The 500 herds in this dataset were originally selected in 2010 from herds in UK that routinely milk record with National Milk Records (NMR). The criteria for selection were that the herds were predominantly comprised of black and white breeds (Holstein, HF, Friesian) and already had good quality monthly milk recording data for a minimum of two years. Herds meeting these criteria were selected using random numbers to ensure a representative cross-section of herds. Following the initial random selection carried out in 2010, the same herds have been retained within the sample for each subsequent annual report. However, each year around 10% of the sample either cease recording or fail to meet data quality criteria. To maintain the sample size at 500 herds, these herds are replaced by randomly selecting new herds that meet the required criteria.

### Analytical framework

For this study the 500 herd dataset that was current at the time of analysis (January 2023) was used. The dataset was queried with the SQL query routinely used in InterHerd+ to generate the ‘cow list’ in the ‘cow performance by calving period’ report. The query included filters to restrict output to first lactations only, with calving dates between 1st January to 31st December 2020. This query produced a row of summary data for each cow-lactation of a cohort of cows first calving in the year 2020. Each row included data on milk production, services, conception, next calving date and/or exit date. The source database contained data on services, conception, re-calving or exit up to August 2022, providing a follow-up period of at least 20 months from first calving to determine the final outcome of each first lactation. The output was exported into an Microsoft Excel spreadsheet (S1 Table).

A series of ‘yes/no’ questions were used in the form of a decision tree to assign parity outcomes for the first lactation cattle in the dataset, i.e.: (i) was the cow served?; (ii) did she conceive?, and; (iii) did she re-calve within the herd or exit without progressing to a second parity?. These questions were answered for each cow by querying the cow’s parity record using Excel functions and applying the following criteria: (i) the cow was served if the number of services recorded is greater than zero; (ii) the cow conceived if days post-partum (DPP) at conception is not null, and; (iii) the cow re-calved in the herd if a re-calving date is recorded or the cow exited the herd if an exit date is recorded in the absence of a re-calving date. A further binary variable was created to indicate cows where an exit reason was recorded as ‘DEAD’ to identify cows that left the herd specifically due to fatality on the farm.

Although the following additional exit reasons of ‘CALV’, ‘DRY’ and ‘SERVE’ were recorded in InterHerd+ to indicate if an event around parturition, during the dry period or at time of serving caused cows to exit the herd, these exit reasons were reported but not relied upon to draw conclusions as they were potentially subject to interpretation. Instead, data on milk production, services, conception, next calving date and/or exit dates were used. Various production parameters were compared between groups of cows with different parity outcomes and between groups of cows with different age at first calving (AFC). The analyses of milk production reported here used data generated for ‘standardised’ 305 days of lactation, including kg milk production and weighted fat and protein percentages and weighted average somatic cell count (SCC). InterHerd+ generates 305 day lactation data for all lactations recorded, using any available milk recording data up to 305 DPP, including incomplete and short lactations. To provide a fair basis for comparison of milk production between cows, data from lactations of less than 290 days duration were excluded from analyses of milk production.

Kruskal-Wallis one-way nonparametric analysis of variance [12] was used to compare continuous variables between groups. Dunn’s all-pairwise comparison [13] was used to identify the significant between-group differences with alpha (probability of a type I error) set at 0.05. The Chi square test was used to compare categorical variables between groups, with Yates’ correction applied where appropriate. A multiple comparison of proportions procedure, equivalent to Tukey’s HSD test for multiple comparison of means in the one-way analysis of variance, was used to identify the significant between-group differences with alpha (probability of a type I error) set at 0.05 [14].

## Results

### Parity outcomes for first lactation cattle

**Fig 1:**
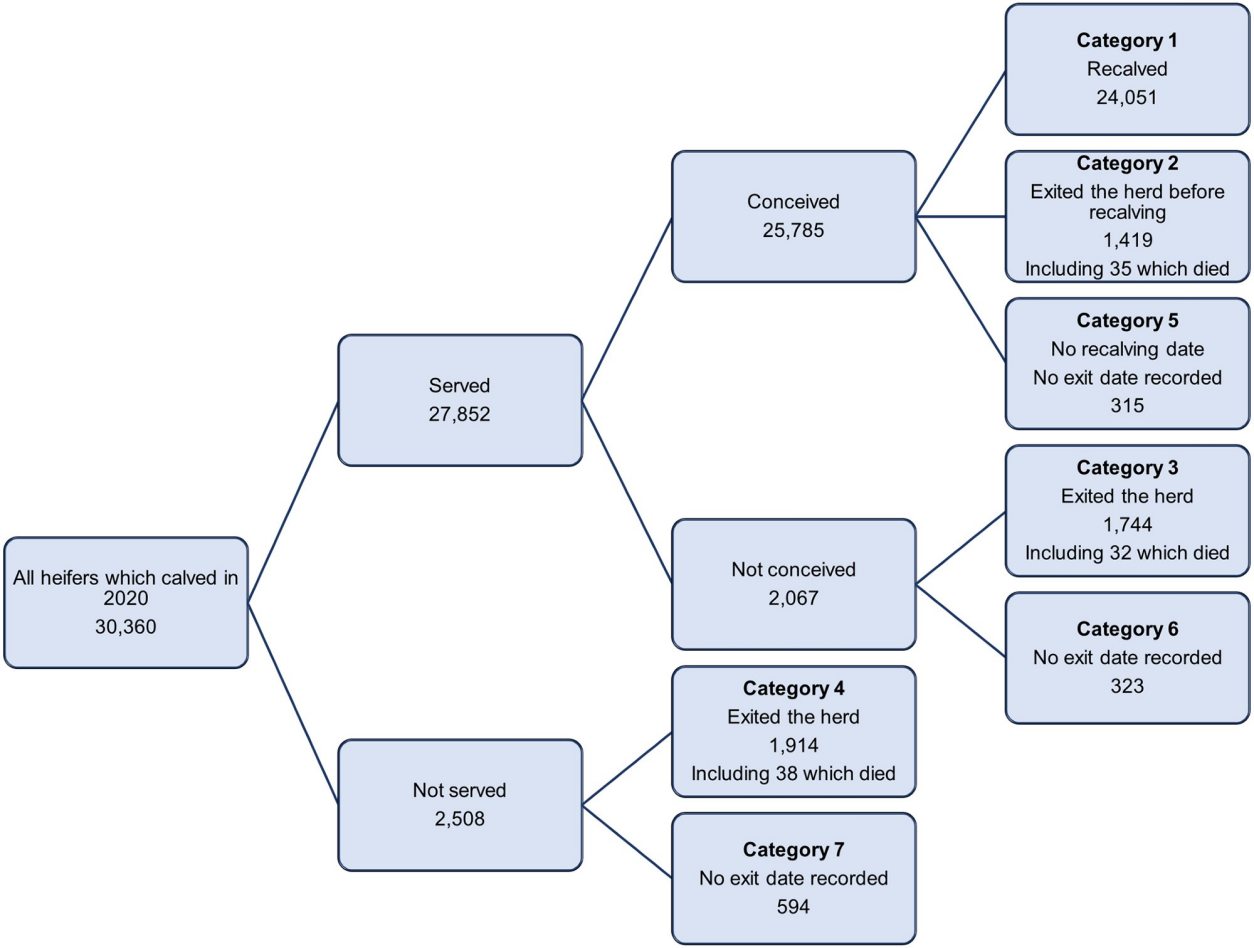
Decision tree to assign parity outcomes for first lactation cows which calved in 2020.

Fig 1 shows the decision tree with the parity outcomes assigned for the 30,360 first lactation cows calving in 2020. The criteria and outcomes are fully tabulated in the S2 Table. The majority (95.9%) of first lactation cows could be assigned to one of four outcomes:

1. re-calved within the herd;
2. exit after a recorded conception date;
3. exit after recorded services but no conception, or;
4. exit with no recorded services or conception

The parity outcome could not be definitely determined for 1,232 (4.1%) of the cows due to the absence of either a re-calve date or an exit date, although all cows had a first calving date at least 20 months prior to data extraction for the study dataset. Because accurate lactation production and fertility statistics could not be reliably calculated for these 1,232 cows they were excluded from further analysis. Details of these excluded cases are provided in S2 and S3 Tables. Table 1 shows the breakdown of the remaining 29,128 cows by parity outcome, along with the number of cows where the exit reason was recorded as ‘DEAD’. Of the first lactation cows with complete records, 82.6% progressed to a second calving within their herds.

**Table 1:** Parity outcomes of 29,128 first lactation cows calving in 2020 (excluding cows with unknown outcomes).

Parity outcome	n (% of total)		n died
1. Re-calved	24,051	(82.6%)	0
2. Conceived: EXIT	1,419	(4.9%)	35
3. Served not conceived: EXIT	1,744	(6.0%)	32
4. Not served: EXIT	1,914	(6.6%)	38
Overall	29,128	(100.0%)	105

### Exits from the herd

Exiting the herd without being served was the most frequent outcome (38% of exits) followed by being served but failing to conceive (34% of exits) and conceiving but exiting the herd before re-calving (28% of exits).

Of 5,077 exits without re-calving, 4,950 recorded an exit reason of ‘CULL/SOLD’ with no further detail. One hundred and five cows were reported to have died: an overall first lactation fatality risk of 0.4% (four in a thousand). The exit reasons recorded for the remaining 22 of the 5,077 exits were: ‘CALV’ (12), presumably sold as soon-to calve cows in advance of a calving date; ‘DRY’ (9) and ‘SERVE’ (1), which could be barren culls. It was also noted that 14 of the cows with a re-calved date were recorded as sold immediately after re-calving, with an exit date the same as their re-calve date, presumably sold as fresh calved cows.

**Fig 2:**
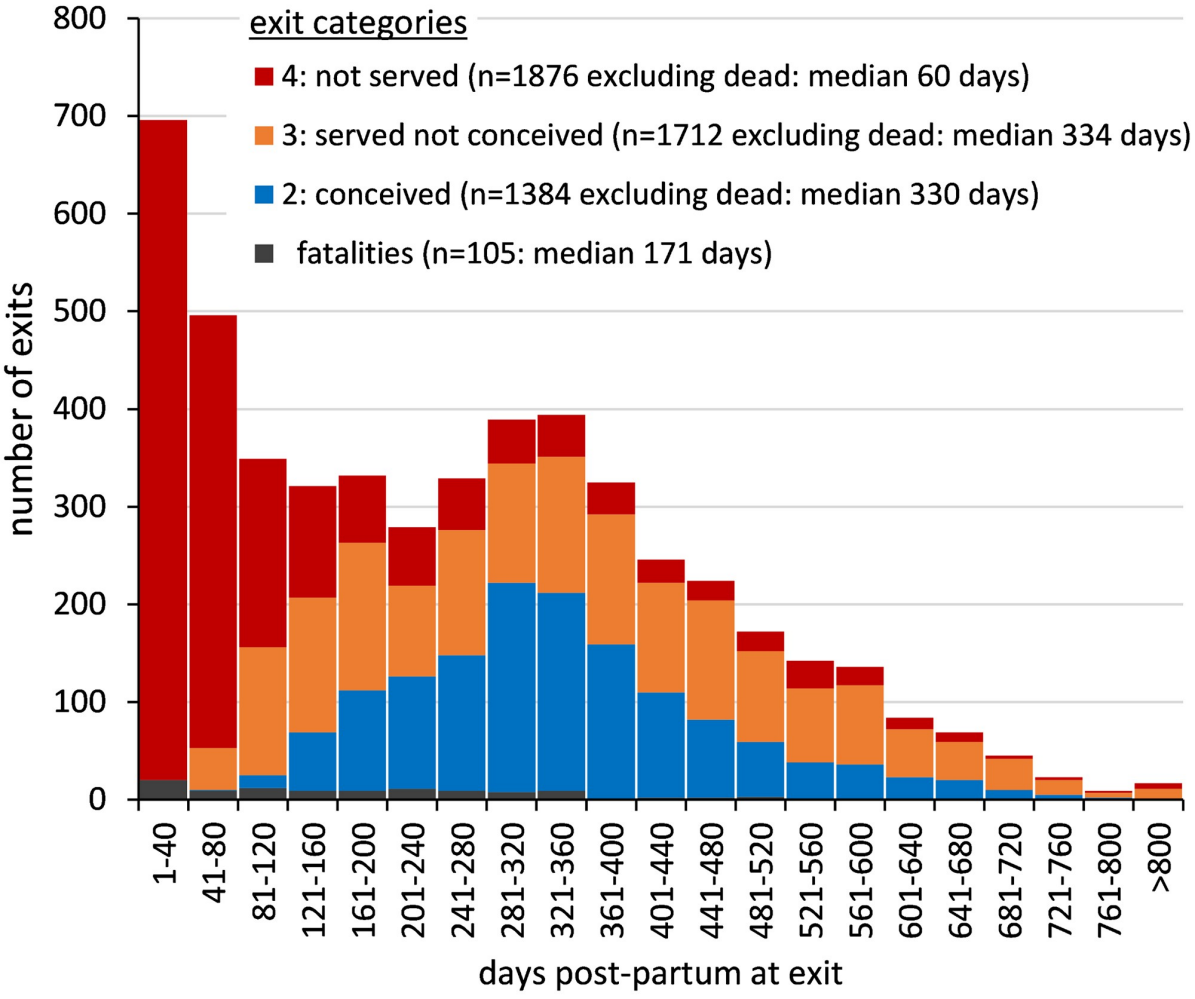
Distribution of cow exits by days post-partum (DPP), showing the different exit categories.

Fig 2 shows the distribution of cow exits by DPP with stacked bars indicating the different exit categories. The 105 fatality exits are shown as a separate data series (regardless of fertility status) and the series for exits with parity outcomes 2, 3 and 4 exclude those fatalities. The distribution of cow exits by DPP is bimodal, with the largest peak in the first 40 DPP and a lower peak between 281 and 360 DPP. In other words, cow exits are most likely to occur soon after calving or around the end of lactation. The early peak is made up almost exclusively of non-served cows, plus some fatality exits. The later peak around the time when most cows would be reaching the end of lactation is made up 53% by cows that have a conception date recorded, 33% served but not conceived, 11% not served, and a few fatalities.

**Fig 3:**
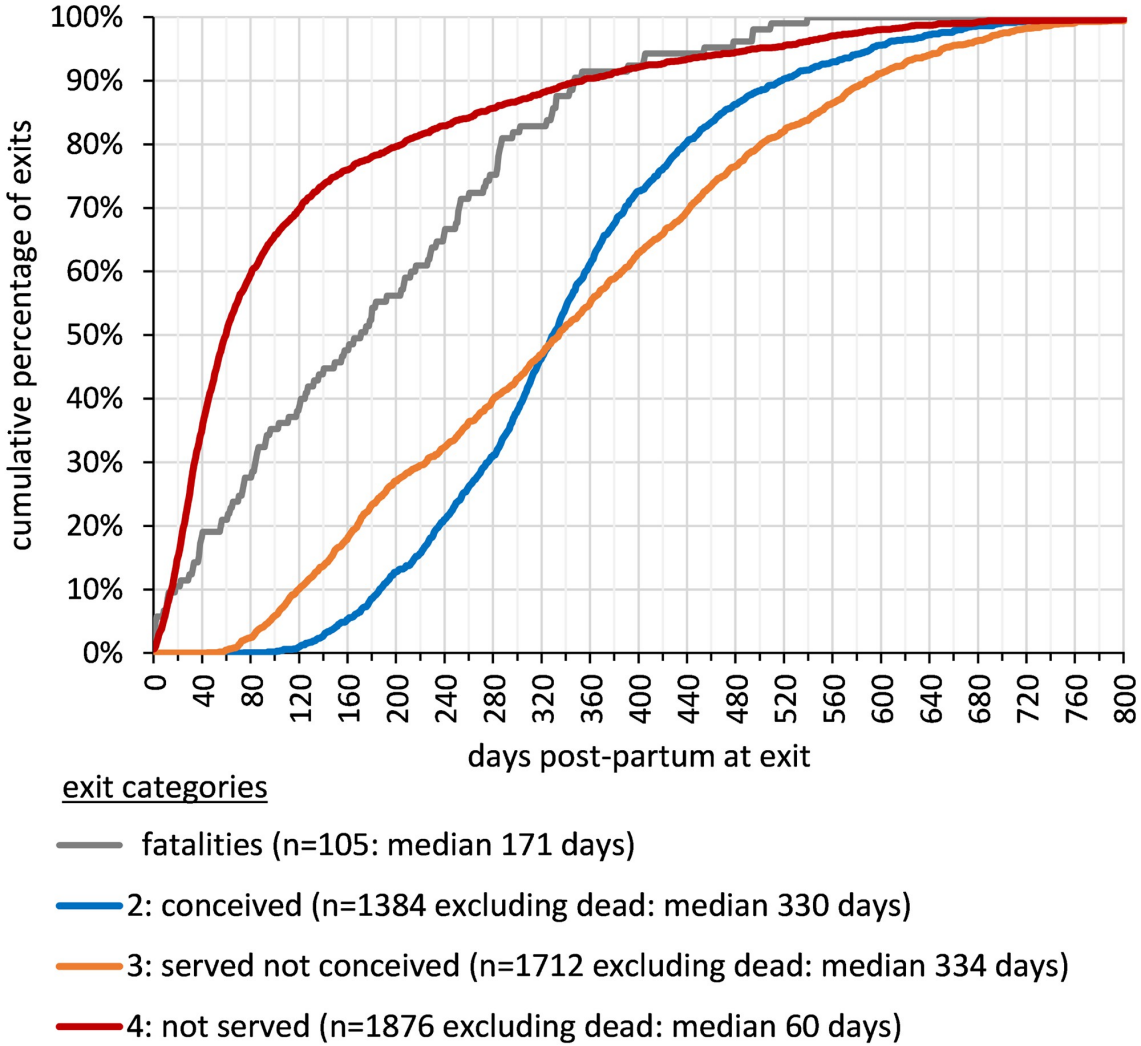
Cumulative relative frequency of cow exits by days post-partum (DPP), shown for different exit categories.

Fig 3 shows the cumulative relative frequency of exits by DPP to more clearly differentiate the distributions of DPP on exit for the different exit categories. Most of the first lactation cattle exiting the herd without being served (outcome 4) were early lactation exits, with 60% of these exits occurring within 80 days after calving increasing to 80% within 200 days after calving. The cumulative relative frequency of exit DPP for cows that were served but did not conceive (outcome 3) is approximately a constant gradient. Approximately 0.5% of exits occurred before 60 DPP after which around 10% of the total exits in this category are disposed of every 60 days, with 91% of these exits accounted for by 600 DPP. The cumulative relative frequency of exit DPP for cows that conceived before exit (outcome 2) is sigmoidal, with hardly any exits before 100 DPP and only 13% of exits occurring within 200 DPP. The frequency of exits is maximum between 280 and 360 DPP with 30% of exits occurring during this period. For cows removed after conceiving it was possible to calculate the interval between the recorded DPP at conception and DPP at exit. Considering a reasonable upper limit gestation of 285 days and an assumed target dry period of up to 65 days the interval when surplus pregnant cows might be sold as ‘near calving cows’ would run from 220 days to 285 days after conception. Thirty percent of the conceived cows exited within this interval, with 47% of exits before the assumed target dry date and 23% exiting over 285 days after the recorded conception date without having calved.

After an initial period of higher death rate with 19% of deaths occurring within 40 days after calving, fatalities were spread evenly over the lactation period up to 360 DPP, by which time over 90% of fatalities had been recorded, with the remainder spread over the subsequent 180 days.

### Fertility performance

Table 2 shows the median values of cow age and DPP at the important events in the fertility cycle: first calving, and then for the first lactation period, first service, conception, end of lactation (dry-off or exit, whichever sooner) and re-calving (for those cows that were retained). Table 2 also shows details of services and conception rates for the cows with different parity outcomes.

**Table 2:** Median age at first calving (AFC) and median days post-partum (DPP) at the important events in the fertility cycle, and other fertility parameters for cows with different parity outcomes.

		Parity outcomes				Overall
		1	2	3	4	
		Re-calved	Conceived: EXIT	Served not conceived: EXIT	Not served: EXIT	
n		24,051	1,419	1,744	1,914	29,128
Medians	AFC (days)	784 ^a^	780 ^a^	800 ^b^	818 ^c^	787
	First service DPP	65 ^a^	66 ^a^	70 ^b^	-	66
	Conception DPP	91 ^a^	97 ^b^	-	-	92
	Dry / Exit DPP	321 ^a^	303 ^b^	318 ^c^	56 ^d^	316
	Re-calving DPP	371	-	-	-	371
Mean serves per cow		2.07 ^a^	2.4 ^b^	3.36 ^c^	-	2.17
Conception rates (%)	OVERALL	48.3% ^a^	41.6% ^b^	0.0%	-	43.1%
	1ST services	46.1% ^a^	38.6% ^b^	0.0%	-	42.7%
	2ND services	49.6% ^a^	44.1% ^b^	0.0%	-	45.2%
	3RD services	50.2% ^a^	42.5% ^b^	0.0%	-	44.1%
% Served by 80 DPP		73.4% ^a^	73.9% ^a^	66.0% ^b^	-	72.9%
% Served by 100 DPP		88.5% ^a^	88.1% ^a^	82.5% ^b^	-	88.1%
% Conceived after 1 serve		46.1% ^a^	38.6% ^b^	-	-	45.7%
% Conceived after 2 serves		72.8% ^a^	65.7% ^b^	-	-	72.4%

Values with the same superscripts across rows are **not** statistically different: probability of type I error (alpha) >0.05

#### Age at first calving (AFC)

Table 2 shows that cows which were successfully re-bred compared to those that left the herd without conceiving or being served were younger at first calving. Kruskal-Wallis one-way nonparametric analysis of variance with Dunn’s all-pairwise comparison (alpha 0.05) showed three distinct parity outcome groups with significantly different AFC:

1. Cows that were served, conceived and then either sold or retained to re-calve (outcomes 1 or 2) had similar AFC distributions, with median AFC values of 780 and 784 days, respectively;
2. Cows that were served but did not conceive (outcome 3) were older at first calving compared to cows that conceived (outcomes 1 or 2), with a median AFC of 800 days, 16–20 days greater than the median AFC for those that conceived;
3. Cows that were not served (outcome 4) were older at first calving than the cows that were served (outcomes 1–3), with a median AFC of 818 days, 18 days greater than cows which were served but not conceived (outcome 3) and 34–38 days greater than cows that subsequently conceived (outcomes 1 and 2).

#### Service and conception for cows with different parity outcomes

**Fig 4:**
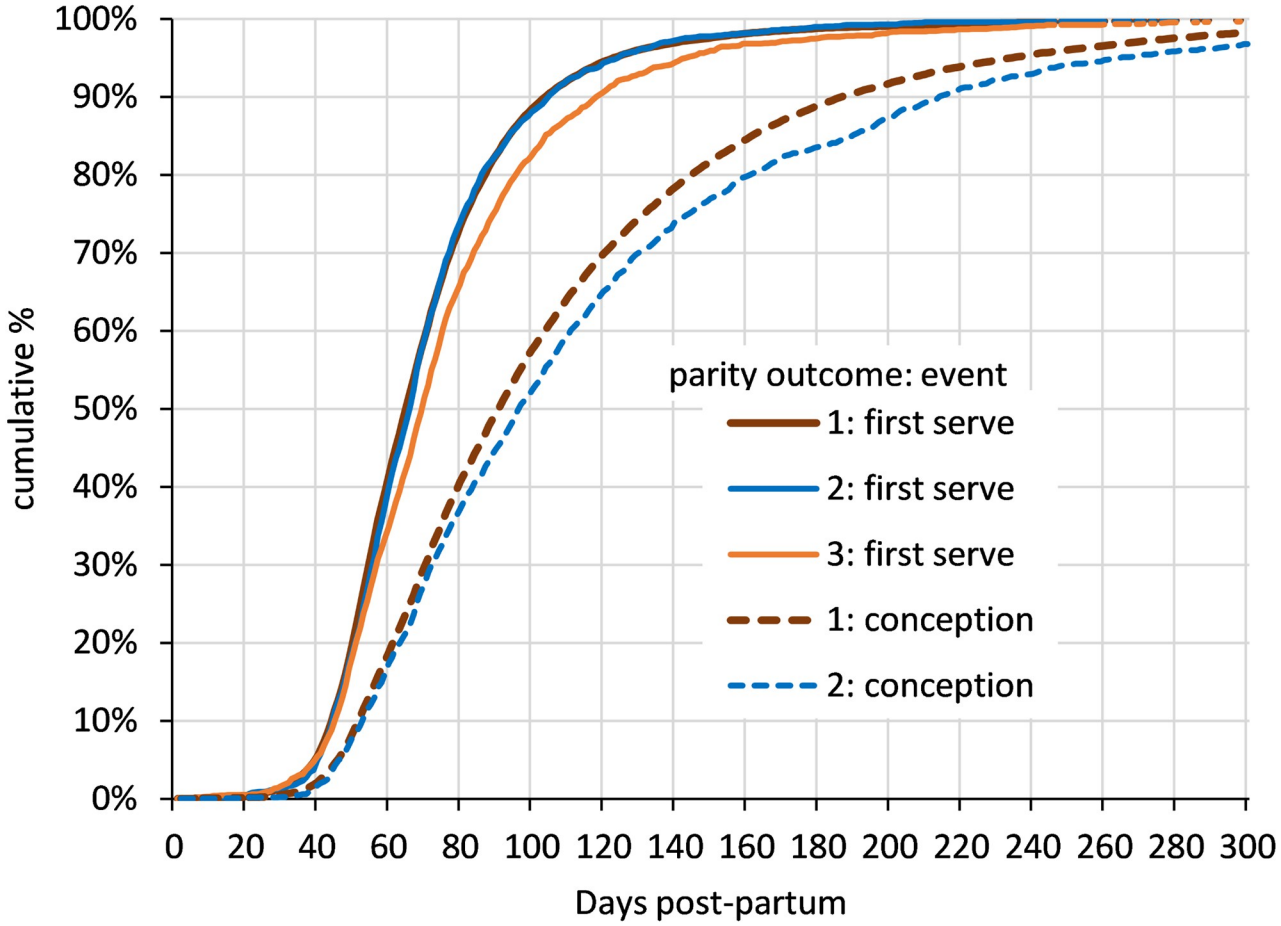
Cumulative relative frequencies of days post-partum (DPP) for cows with different parity outcomes. The distribution of first service and conception (where applicable) are shown for cows within parity outcomes 1 to 3.

Fig 4 shows the cumulative relative frequencies of DPP at first service and DPP at conception for cows with different parity outcomes. Fig 4 and Table 2 show that first service was later for cows that were eventually disposed of without having conceived (outcome 3) compared with cows that conceived (outcomes 1 and 2) that had almost identical medians and distributions of DPP at first service. However conception was later in the cows that were not retained compared with those that re-calved in the herd. Kruskal-Wallis one-way nonparametric analysis of variance with Dunn’s all-pairwise comparison (alpha 0.05) confirmed significantly different DPP at first service between the two outcome groups that conceived combined and the group that were disposed of without having conceived (outcomes 1 and 2 *versus* outcome 3) and also significantly different DPP at conception between the two outcome groups that conceived (outcome 1 *versus* outcome 2).

With respect to commonly used target service and conception time limits, Table 2 shows similar percentages of cows that conceived (outcomes 1 and 2) were served by 80 and 100 DPP (around 73%/74% at 80 DPP and 88% at 100 DPP). Of the cows that were served but did not conceive (outcome 3) only 66% and 82% had been served at 80 and 100 DPP, significantly lower percentages than the cows that conceived (Chi square, p<0.0001 in both cases).

Cows that conceived and were retained to start a second parity (outcome 1) displayed better conception rates (overall, first service, second service and third service) than the cows that conceived but were not retained (outcome 2). All differences between the two groups were statistically significant (Yates’ corrected Chi square, p<0.01). As a result, higher percentages of the retained cows had conceived after 1 and 2 serves than the cows that conceived but were not retained (Chi square, p<0.0001 in both cases) (Table 2).

First lactation cows with the different parity outcomes had received different numbers of services on average. Cows that conceived and were retained to start a second parity (outcome 1) were served fewer times on average (mean 2.07 services per cow); cows that conceived but were not retained (outcome 2) had more service attempts on average (mean 2.40 services per cow); cows that were served but were disposed of without conceiving (outcome 3) had received the most service attempts on average (mean 3.36 services per cow) (Table 2). Kruskal-Wallis one-way nonparametric analysis of variance with Dunn’s all-pairwise comparison (alpha 0.05) showed that all the three means were significantly different from one another.

#### Timeline for first lactation cattle which remain within the herd and re-calve

**Fig 5:**
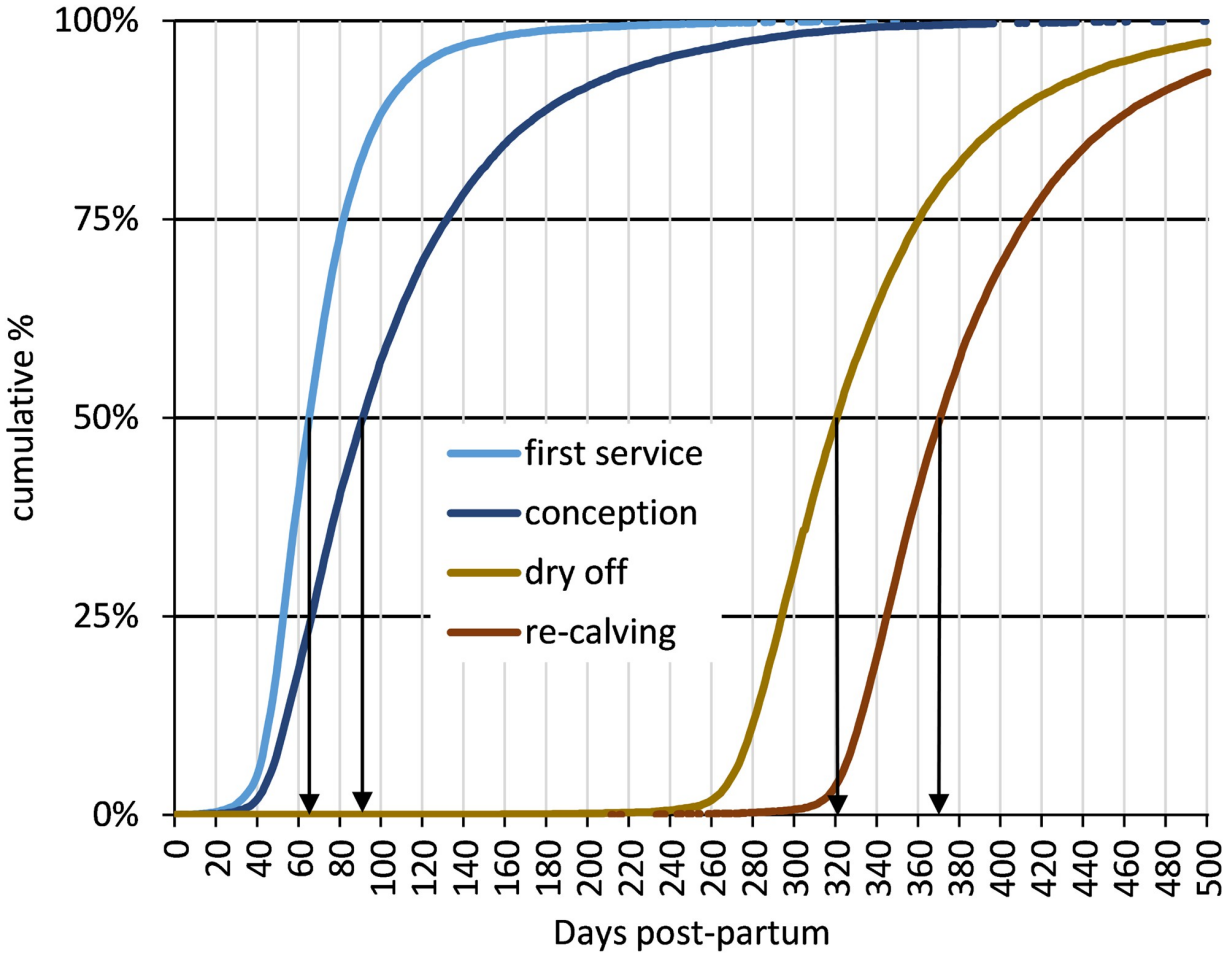
Distributions of fertility events for cows which progressed to 2nd lactation within the same herd.

Fig 5 shows the distributions of fertility event timings (DPP) for first lactation cows which progressed from 1st to 2nd lactation within the same herd. Median values for the key reproductive cycle stages for these cattle were 784 days (about 25 months and 21 days) of age at 1st calving, 65 DPP for 1st service, 91 DPP for conception with a median lactation length of 321 days and calving interval of 371 days (Table 2). On average, these cattle required 2.07 services per conception with overall and 1st service conception rates of 48.3% and 46.1%, respectively (Table 2). There is some variation in DPP at first service and more variation in DPP at conception, with an interquartile range of 66 days. This results in an inter-quartile range of 68 days for the re-calving interval between 345 DPP and 413 DPP (Fig 5).

### Production performance

Table 3 shows mean and median 305 day milk production (kg), weighted fat and protein percentages and weighted average SCC (cells/ml x10^3^) for first lactation cows with different parity outcomes, excluding lactations of less than 290 days recorded duration. Kruskal-Wallis one-way nonparametric analysis of variance shows significant differences in milk yield between cows with different parity outcomes (p = 0.0002). However, Dunn’s all-pairwise comparison (alpha 0.05) identified the only significant difference as being between cows that conceived (outcomes 1 and 2) and cows that were never served (outcome 4), with mean yield about 500kg lower in not served cows. It is important to note that most of the cows with outcome 4 exited the herd before 290 DPP, so that only 228 of the 1,914 outcome 4 cows were included in this analysis. Kruskal-Wallis one-way nonparametric analysis of variance revealed no significant differences in fat and protein content between cows with different parity outcomes (fat %, p = 0.7; protein%, p = 0.8). Kruskal-Wallis one-way nonparametric analysis of variance shows significant difference in SCC between cows with different parity outcomes (p<0.0001). Dunn’s all-pairwise comparison (alpha 0.05) identified the significant difference as being between cows that conceived (outcomes 1 and 2) and cows that did not conceive or were never served (outcomes 3 and 4), with a median weighted average SCC being 11,000 to 13,000 cells/ml higher in not served cows.

**Table 3:** Mean and median 305 day lactation statistics for cows with different parity outcomes (excluding data from lactations of less than 290 days duration).

	Parity outcomes				Overall
	1	2	3	4	
	Re-calved	Conceived: EXIT	Served not conceived: EXIT	Not served: EXIT	
n Qualifying Lactations*	18,284	820	979	228	20,311
	Mean: Median	Mean: Median	Mean: Median	Mean: Median	Mean: Median
Prod305 (kg)	8,261: 8,167 ^a^	8,383: 8,335 ^a^	8,174: 8,030 ^ab^	7,832: 7,634 ^b^	8,257: 8,165
AvProt (%)	3.3%: 3.3%	3.3%: 3.3%	3.3%: 3.3%	3.3%: 3.3%	3.3%: 3.3%
AvFat (%)	4.2%: 4.2%	4.2%: 4.2%	4.2%: 4.2%	4.1%: 4.2%	4.2%: 4.2%
AvSCC (cells/ml x 10^3^)	104: 45 ^a^	95: 43 ^a^	130: 52 ^b^	169: 56 ^b^	106: 45

* Note: cows with short lactations (<290days) were excluded

Values with the same superscripts across rows are **not** statistically different: probability of type I error (alpha) >0.05

### Comparison of fertility and production parameters in cows with different AFC

Table 4 shows the percentages of cows which were served, conceived, re-calved, exited the herd or died, alongside other fertility parameters and 305 day lactation statistics, for cows with different AFC.

**Table 4:** Proportion (%) of cows served, conceived, re-calved, exited the herd or died, with other fertility parameters and 305 day lactation statistics, for cows with different age at first calving (AFC).

	AFC(m) group				
	<23m	23m-25m	26m-28m	29m-31m	>31m
n Cows	3,062	12,210	7,499	3,278	3,079
% Served	94.2% ^a^	95.1% ^a^	93.0% ^b^	91.4% ^c^	89.4% ^d^
% Conceived	88.3% ^a^	89.8% ^a^	86.6% ^b^	84.7% ^bc^	82.3% ^c^
% Re-calved	82.9% ^a^	84.8% ^a^	81.7% ^b^	79.8% ^bc^	78.4% ^c^
% Exited	17.2% ^a^	15.2% ^a^	18.4% ^b^	20.2% ^bc^	21.7% ^c^
% Died	0.26% ^a^	0.26% ^ab^	0.41% ^ab^	0.49% ^ab^	0.58% ^b^
n Cows served	2,884	11,612	6,972	2,995	2,751
% Conceived of those served	93.7% ^a^	94.4% ^a^	93.1% ^b^	92.7% ^b^	92.2% ^b^
Median DPP to…					
…First service	65 ^a^	65 ^a^	64 ^b^	69 ^c^	73 ^d^
…Conception	87 ^a^	90 ^a^	90 ^b^	96 ^c^	101 ^d^
Mean serves/cow	2.21 ^a^	2.17 ^a^	2.22 ^a^	2.10 ^b^	2.11 ^b^
Conceptions / all services (%) (conception ‘rate’)	42.4% ^b^	43.6% ^b^	41.9% ^a^	44.1% ^b^	43.8% ^b^
% Re-calved of those conceived	93.9% ^a^	94.5% ^a^	94.4% ^a^	94.3% ^a^	95.2% ^a^
**305 Day lactation statistics (excluding data from lactations of less than 290 days duration)**					
n Qualifying Lactations	2,125	8,583	5,133	2,328	2,142
	mean: median	mean: median	mean: median	mean: median	mean: median
Prod305 (kg)	8,494: 8,412 ^a^	8,348: 8,251 ^a^	8,326: 8,244 ^b^	8,024: 7,991 ^c^	7,750: 7,672 ^d^
AvProt (%)	3.3%: 3.3%	3.3%: 3.3%	3.3%: 3.3%	3.3%: 3.3%	3.4%: 3.3%
AvFat (%)	4.1%: 4.1%	4.2%: 4.2%	4.2%: 4.2%	4.2%: 4.2%	4.2%: 4.2%
AvSCC (cells/ml x 10^3^)	108: 42 ^a^	99: 43 ^b^	107: 46 ^b^	112: 48 ^b^	119: 53 ^c^

Values with the same superscripts across rows are **not** statistically different: probability of type I error (alpha) >0.05

#### Fertility in first lactation (all cows)

Table 4 shows the percentages of cows which were served, conceived and re-calved consistently decline with AFC 26 months and above, with corresponding increases in the percentage of cows exiting the herd. In particular:

- The percentage of cows served declined from 95% in the 23–25 months AFC group to 89% in the >31 months AFC group (overall Chi square p<0.0001). The multiple comparison of proportions test showed that the proportion of cows served in all groups, except the <23 months AFC group, was statistically significantly different (p<0.05). The <23 months AFC group was not statistically significantly different from the 23–25 months or 26–28 months AFC groups.
- The percentage of all cows that conceived declined from 90% in the 23–25 months AFC group to 82% in the >31 months AFC group (overall Chi square p<0.0001). The multiple comparison of proportions test showed that the proportion of cows which conceived within the 23–25 months AFC group was statistically significantly different (p<0.05) from all other groups, except the <23 months AFC group. The <23 months AFC group was not statistically significantly different from the groups with an AFC of 23–25 months or 26–28 months (p> = 0.05). Also, the differences between the 26–28 months and 29–31 months AFC groups and between the 29–31 months and >31 months AFC groups were not statistically significantly different (p> = 0.05). However, when looking at only the cows that were served (shown as a separate row in Table 4) there was a smaller difference between the 23–25 months (94% conceived) and >31 months AFC groups (92% conceived) (overall Chi square p<0.0001). The multiple comparison of proportions test identified that the proportion of cows which conceived within the 23–25 months AFC group as statistically significantly different (p<0.05) from all other AFC groups, except the <23 months group, while differences between all other groups were not statistically significantly different (p> = 0.05).
- The percentage of cows that re-calved within the herd declined from 85% in the 23–25 months AFC group to 78% in the >31 months AFC group (overall Chi square p<0.0001). The multiple comparison of proportions test identified the same pattern of differences between groups as for the percentage conceived. However, when looking at only the cows that conceived (shown as a separate row in Table 4) the percentages that were retained and re-calved did not vary greatly with AFC (between 94% and 95% of conceived were retained) and these differences were not statistically significant (overall Chi square p = 0.3566).
- The percentage of cows exiting the herd without re-calving was a mirror-image of the percentage of cows that re-calved within the herd, increasing from 15% in the 23–25 months AFC group to 22% in the >31 months AFC group (overall Chi square p<0.0001).
- The percentage of that cows died increased from 0.26% in the <23 months and 23–25 months AFC groups to 0.58% in the group with AFC >31 months (overall Chi square p = 0.0327). While the overall Chi square was statistically significant (p<0.05), the absolute numbers of cows recorded as died were small and the multiple comparison of proportion test only identified a statistically significant difference between the 23–25 months and the >31 months AFC groups.

Looking at the detailed fertility parameters of those cows that were served in particular:

- The median DPP at first service varied from 64 days in the 26–28 months AFC group to 73 days in the >31 months AFC group. Kruskal-Wallis one-way nonparametric analysis of variance showed significant differences in DPP at first service between cows with different AFC (p<0.0001). Dunn’s all-pairwise comparison (alpha 0.05) identified all groups as being different from each other except between the <23 months and the 26–28 months AFC groups. However, the absolute differences between the groups up to 28 months AFC were small, with no more than a day between the medians.
- The median DPP at conception varied more or less in parallel with the median DPP at first service, with a Kruskal-Wallis p-value <0.0001. The number of services per cow were similar across the AFC groups although the Kruskal-Wallis one-way nonparametric analysis of variance indicated some significant differences (p = 0.0002), with Dunn’s all-pairwise comparison (alpha 0.05) showing that the main difference were between the <23 months to 28 months AFC groups and the > 28 months AFC groups.
- The overall conception rate to all services did not vary much with AFC although the Chi square test indicated some significant variation between AFC groups (p = 0.0017). The multiple comparison of proportions test identified the main significant difference as being between the 26–28 months AFC group and the groups either side.

#### Production in first lactation

Table 4 shows mean and median 305 day milk production (kg), weighted fat and protein percentages and weighted average SCC (cells/ml x10^3^) for first lactation cows with different AFC, excluding lactations of less than 290 days recorded duration. In particular, Kruskal-Wallis one-way nonparametric analysis of variance showed significant differences in milk yield between cows with different AFC (p<0.0001), with milk yield declining as AFC increased. Dunn’s all-pairwise comparison (alpha 0.05) identified that all groups were different from each other except the 23–25 months AFC and 26–28 months AFC groups. The biggest difference between mean 305 day milk production was about 750 kg between the <23 months and >31 months AFC groups.

Kruskal-Wallis one-way nonparametric analysis of variance showed significant differences in % protein and % fat between cows with different AFC (p<0.0001) although differences must be small since the means and medians both appeared similar across the AFC groups when rounded to 0.1%.

The median of AvSCC increased above 25 months AFC, with differences in median weighted average SCC of up to 10,000 cells/ml between the 23–25 months and the >31 months AFC groups. Kruskal-Wallis one-way nonparametric analysis of variance showed significant differences in SCC between cows with different parity outcomes (p<0.0001). Dunn’s all-pairwise comparison (alpha 0.05) identified three distinct AFC groups: ≤25 months; 26 to 31 months and >31 months.

## Discussion

Data from 500 dairy herds has been used to monitor UK annual cull rates since 2010 [10]. However, literature on the retention or removal of cows in their first lactation is limited. Identifying the factors influencing UK farmer decisions to remove first lactation cattle from the herd could promote mitigating measures, leading to an increased proportion of first lactation cows progressing to second lactation and reduction in over-supply of replacement heifers. Milk recording data from the 500 dairy herds was used to determine the proportion of first lactation cows which progress to second lactation in the same herd and the full breadth of associated milk recording data was used to explore factors potentially influencing farmer decisions to keep or remove first lactation cattle from the herd.

The headline result of this analysis was that 82.6% of first lactation cows were retained into second lactation with a median calving interval of 370 days. This means that 17.4% of first lactation cattle were removed from the herd before re-calving. A previous examination of first lactation cows in the UK noted that a similar though slightly higher proportion, namely 19%, of cows exited the herd in their first lactation [2]. This current finding compares favourably with estimates from other countries with intensive dairy systems. In Estonia, 16% of first lactation cows were culled in their first lactation [6], slightly lower than the proportion found here. In contrast, some countries have found much higher proportions of first lactation cows culled in their first lactation: as high as 28% in Sweden and 29% in the USA [3, 4]. The exit reasons were broadly similar between countries. Within the UK, of the first lactation cows which were culled, 37% were culled due to infertility [2]. In Estonia, of the first lactation cows which did not progress to second lactation, 25% were culled due to feet/claw disorders, 18% due to udder disorders and 15% due to fertility problems [6]. In the Swedish study, poor fertility (conventional, 32% of culls; organic, 31% of culls), udder health (conventional, 15%; organic, 21%) and low milk production (9% of culls in both types) were the most common reasons given for first lactation cows not progressing to second lactation [4]. The distribution of cow exits by DPP within our study was bimodal, with the largest peak in the first 40 DPP and a lower peak between 281 and 360 DPP. This matches findings of Hadley et al. [15] who noted two cull peaks at the beginning and end of lactation. A small number of the exits were recorded as on-farm deaths giving a fatality risk of 0.4%. These fatalities were recorded for cows in all the three fertility states described in the results and the distribution of DPP on exit for these fatalities suggests a range of fatality causes affecting cows at any stage of the fertility / lactation cycle, although there was slight bias towards fatality in the first 40 DPP. In addition to these specifically recorded deaths, non-fatal but serious accidents or conditions requiring immediate on-farm euthanasia. could have contributed to exits of cows with any fertility status, but the data contained no details that could be used to characterise this number.

The 17% of first lactation cows that did not re-calve within their herds could be classed as ‘wastage’. However, our milk recording data detailed if and when first lactation cows left the herd, but not if these cows were culled (i.e. slaughtered) or were sold to continue their productive life on another farm. Encouragement from the UK milk recording organisations to farmers to collect detailed exit information would allow the most efficient longevity of dairy cows to be more easily calculated would allow the most efficient longevity of dairy cows to be more easily calculated. Canada [16], Austria [17], Denmark, Finland, Norway and Sweden have setup a central database which combines milk recording data and health data [18]. A combined milk recording and health database would allow the exit reasons for first lactation cows to be easily identified. Nevertheless, milk recording data detailing if and when first lactation cows left the herd considered alongside the timing of removal (DPP at exit), fertility status on exit can provide clues about farmers’ decision making about removal. Fertility data recorded alongside the milk recording data allowed us to distinguish cows leaving the herd in different states of fertility, specifically: 38% of exits had no recorded services or conception; 34% of exits had been served but not conceived, and; 28% of exits had been served and had conceived.

### Exits in different states of fertility

#### Exit with no recorded services or conception

The majority of first lactation cows which exited the herd with no recorded services or conception exited within early lactation (Fig 2) when cows are more likely to experience post-partum health disorders. The most common postpartum health disorders (and their prevalence) in first lactation cows are subclinical mastitis (20.7%), metritis (17.1%), subclinical hypocalcemia (17.1%), clinical mastitis (7.6%), retained fetal membranes (6.4%), subclinical ketosis (6.1%), clinical ketosis (4.0%), clinical hypocalcemia (0.9%) and displaced abomasum (0.9%) [19]. Considering cows of all parities, 61% and 25% of cows are diagnosed with ≥ 1 or ≥ 2 health disorders, respectively [19]. Health data was unavailable but cows which exited with no recorded services or conception are likely to have been diagnosed with ≥ 1 health disorders. These cows could also have been lame, within England and Wales the mean within farm lameness prevalence is 31.6% [20]. Lameness is more common is early lactation [21] but can affect cows at any stage of lactation so it likely a reason for exit across all parity outcomes. A small proportion of non-served exits could also have been due to non-fatal accidents or conditions related to calving, requiring casualty slaughter [2, 3]. Other reasons for first lactation cows not being served and then disposed of, particularly after a longer time post-partum, could include failure to show heat or unsuitability for retention in the milking herd for other reasons such as temperament or sub-optimal growth [2, 3]. Nevertheless, a large proportion of these non-served exits were presumably judged surplus to requirement. A farmer may have reared more heifers than eventually needed [22] as contingency against unpredictable production-related culling or enforced culling, as a result of bTB testing for example [23]. The farmer would then face the decision of either removing an older cow, or disposing of the first lactation cow. These surplus first lactation cows could have been sold as ‘fresh-calved cows’ soon after calving and could be destined as dairy replacements in other herds. In the studied cohort, 60% of non-served exits were disposed of within 80 DPP and it is not unreasonable to suggest that some of these cows may well have gone on to a second lactation in other herds. Thus, the majority of disposals without services could be the result of decisions taken by the farmer soon after first calving, if not before. While the data showed a significantly lower milk yield, with higher SCC, in cows that were never served compared with cows that conceived, this information would not be available to the farmer at the time the decision not to serve would be taken. However, AFC would be available at that time and could be a factor in farmers’ decision making, apparently favouring serving of cows with the younger AFC. In the data studied here, the cows that were disposed of without serving had a median AFC which was 18 days greater than cows served but not conceived and 34–38 days greater than cows served and subsequently conceived.

#### Exit after recorded services but no conception

Poor fertility is logically the main reason for removals of cows with recorded services but no conception. The vast majority of these exits will be culls, although some could conceivably be bought as potential breeders by other herds if they are sold early in lactation. For comparison, a previous study in UK noted that 37% of first lactation culls were due to infertility [2] and studies in Poland, Sweden and Estonia reported between 15% and 44% of first lactation culls due to infertility [5, 6, 9]. The exact reason for the discrepancies between countries is unclear. However, it is likely related to different management policies on heifer rearing, the availability of replacements and cut-off at which farmers believe poor fertility performance warrants a cow be removed from the herd. In the cohort studied here, the time that farmers allowed for the first lactation cows to conceive before exiting the herd varied widely, with a fairly even distribution of DPP at exit in this group. Approximately 0.5% of exits occurred before 60 DPP after which around 10% of the total exits in this category were disposed of every 60 days. This group also received at least one more service per cow on average than cows that were served and conceived, so in general these were cows that the farmer was trying to re-breed. The cow exits in this category could be described as an attrition of cows that are failing to conceive mixed with some cows that ‘fail’ for other reasons. Exits in this category with only one or two services could have had poor heat expression or irregular cycles or developed serious mastitis or other health problems.

The data revealed that the cows served but not conceived tended to have first-calved older than the cows that conceived, with a median AFC 16–20 days greater than the median AFC for those that conceived, and also had a later median DPP at first service than those that conceived. This may be an indication of sub-optimal fertility among these cows, both in terms of delayed conception as heifers and delayed return to normal oestrus after first calving.

#### Exit after a recorded conception date

There could be a variety of reasons for removing first lactation cows subsequent to a recorded conception date. The data analysed here showed no significant difference in 305 day milk yield or average SCC between the conceived cows that were removed and those retained, with the means and median values of both parameters in fact being slightly ‘better’ in the removed group. However, the conceived but removed cows had slightly inferior fertility parameters compared to the cows retained into second lactation. Despite no significant differences between DDP at first service the cows that were removed had a lower conception rate to all services and therefore conceived later and require more services to conceive, with a median DPP at conception of 97 days compared with 91 days. It therefore appears that timely conception to maintain a tight calving interval took priority over milk yield and quality when deciding which pregnant first lactation cows to remove. According to the interval between DPP at conception and DPP at exit, 30% of these cows could have been sold as ‘near calving cows’ after a full lactation. Forty seven percent were sold earlier in lactation, perhaps due to health issues or management decisions related to cash flow, lack of feed or space. The remaining 23% were sold at more than 285 days after the recorded conception day without having calved, so these could be the result of an abortion [2], false positive pregnancy diagnosis or lost pregnancy through unnoticed or unrecorded abortion.

### AFC as a factor influencing retention or removal of first lactation cows

The data revealed younger median first calving ages for cows that were successfully re-bred, whether subsequently retained or not, compared to those that left the herd without conceiving or being served. When considering the cows grouped according to AFC in months, the proportions which were served, conceived and re-calved were highest and proportion of cows which exited the herd was lowest in the group with an AFC of 23 to 25 months. There were some differences in days to first service and conception rates between the AFC groups, with the cows with AFC up to 25 months performing best, but the main difference was in the percentage that were served, decreasing with increasing AFC. The lower percentage of cows with a higher AFC re-calving therefore appears to be mainly due to a management decision not to serve, rather than a physiological fertility issue. This possibly reflects the case that farmers fill up their replacement places by serving the heifers that calve younger, and possibly earlier in the calving season. The heifers that calve older and later in the calving season are more likely to be judged as ‘spare’ and therefore not served, unless they have some other special attribute the farmer wants to keep.

Milk production (kg) declined and average SCC generally increased with increasing AFC. The mean 305 day yield was about 750 kg lower in the group with AFC greater than 31 months than the group with AFC less than 23 months. There were significant differences in median weighted average SCC, being 10,000 cells/ml greater in the group with AFC greater than 31 months than the group with AFC 23–25 months. If these are common trends known to farmers, this could be a factor in farmers’ favouring serving cows with younger AFC.

Similar results favouring cows with younger AFC have been reported elsewhere. An analysis of milk records from 7,256 first lactation Jersey cows concluded that ≤24-months is the optimal AFC and that first lactation Jersey cows with an AFC of 30-months were less likely to progress to second lactation [24]. Likewise, milk records from 35,128 first lactation HF cows across 33 herds in Hungary suggested that first lactation cows with an AFC ≤22-months were least likely to be culled within 50 DPP and that the likelihood of first lactation cows progressing to second lactation decreased as AFC increased [25]. In our dataset, 53% of first lactation cows had AFC up to 25 months, with 43% between 23 and 25 months.

### Balancing the breeding of replacement heifers and longevity

Replacement heifers will always be needed in dairy herds. They are needed to replace older cows that the farmer considers to have reached the end of their economically useful productive life, or that have to be removed for fertility, health or welfare reasons.

In our 2020 cohort it appeared that farmers had reared and calved more heifers than were needed because 6.6% of first lactation cows were removed without any attempt to breed. Perhaps this is intentional (to sell for profit) or perhaps reflects over-supply of first calving cows. The available milk recording data could not provide much information as to how farmers might decide which first calved cows to serve and which to not serve. The data showed a tendency to favour serving those with the younger AFC. Other factors were not clear from the data available in this dataset, but could include pedigree/genetics, behaviour (e.g. adaptation to milking parlour / ‘kickers’).

Farmers also served more first lactation cows than are finally retained as herd replacements into second lactation, because 6.0% were removed after failing to conceive and 4.9% were removed despite having conceived. It is rational to serve more first lactation cows than the anticipated number required to maintain the herd because a level of ‘fertility failure’ can be expected (failure to conceive within the timeframe set by the management of the herd) and there is also a background risk of fatalities and production-limiting accidents or health and welfare conditions. Because the level of ‘fertility failure’ and other losses is uncertain, farmers act conservatively when deciding how many first lactation cows to serve. The result found here was that more first lactation cows are successfully bred to conception than were needed to progress to second lactation within their herds. Farmers then have to decide which pregnant first lactation cows to dispose of. The data available in this dataset suggest AFC and DPP at conception are the most important factors that may be considered by farmers, with 305 day yield and average SCC being of minor importance. Overall it could be said that in order to be retained in the herd, cows need to fit into the fertility ‘calendar’ of the whole herd to maintain the management targets for calving interval and calving season. Therefore heifers need to calve as near to 2 years as possible, and then conceive as close to 90 DPP as possible to achieve a 370 day calving interval. Heifers that calve with older AFC are less likely to be served and first lactation cows that conceive later are less likely to be retained.

Over-supply of replacement heifers represents an inefficiency in the dairy supply chain. In current times there is increasing pressure to increase efficiency in the dairy supply chain both from an economic point of view and in order to reduce the carbon footprint of milk production. Farmers have made dramatic improvements in recent years in the fertility, production and health of dairy cows across the UK, removing many of the traditional causes for early culling of animals. However, this has not resulted in greater longevity in dairy cows across the UK. In 2022, the average lactation number and age of culled cows of the median dairy herd in a sample of 500 herds was 0.3 less lactations and 0.6 years younger than in 2010 [10]. Over the same period there have been significant advances in the use of genetics to improve production, fertility, animal welfare, environment and physiology [26]. These benefits are reflected in farm economic performance, Spanish farms with a high genetic index demonstrated higher milk yields (L/cow), milk sale (€/cow) and gross margins (€/cow) compared to herds with a low genetic index [27]. Genetic advances combined with the introduction of sexed semen, have greatly facilitated the supply of replacement heifers of high genetic merit. These advances have worked against any progress to greater longevity as younger cattle benefit from this genetic progress. A herd that produces more replacement heifers is likely to cull older cows to make space in the herd on the basis that ‘*a new cow is better than an old one*’, without adequate consideration of the economic and resource efficiency implications. On top of this, the current study suggests more heifers are reared than needed to support even an increased level of herd turnover, and substantial numbers of first-calved cows are not served, or conceive and are not retained, representing a further source of inefficiency. Increasing the productive life of dairy cows, along with reducing or eliminating over-production of replacement heifers would be a positive way to improve efficiency and reduce the climate impact of dairy production.

The longevity of cows in dairy herds can be extended beyond current levels without detriment to economic output [28]. An economic analysis by De Vries [11] suggested that the optimal productive lifespan of dairy cattle is five lactations, based on one lactation per year, compared to the most recent UK 500 herds study in which the median herd averaged 3.6 lactations for cows exiting the herd [10]. According to Boulton et al. [1] heifers do not payback their rearing costs until they are in their second lactation. Profit per kg of fat and protein corrected milk is positively related to productive lifespan while at the same time lifetime greenhouse gas emissions per kg fat and protein corrected milk (FPCM) is negatively related to productive lifespan [29]. A case study in Germany has shown that increasing the length of productive life of dairy cows is a viable way to reduce the climate impact [30].

If cows are kept in production for more lactations then fewer replacements are needed each year. To fully benefit from keeping older cows, the reduced requirement for replacements needs to be recognised and fewer heifers should be reared, therefore saving heifer rearing costs and avoiding over-supply. When estimating requirements for herd replacements farmers face the difficulty of having to anticipate requirements more than two years in advance, this being the lead time from serving a cow with dairy semen to the first calving of the resultant heifer. An additional problem farmers face in a predominantly seasonal calving management set up is that they have to serve heifers, first lactation cows and older cows at around the same time, so if fertility performance is better than expected are they will have more pregnant cows to potentially retain in the herd than are needed at all ages.

Farmers need support to establish a reliable estimate of the baseline cull rate of cows that ‘break down’ and require culling each year and also to identify cows in the herd as ’voluntary’ cull candidates, based on their performance in the herd and the efficiency trade-off of replacement cost versus ‘new cow benefit’. The use of routine milk recording with monitoring of fertility parameters can help farmers to more accurately understand the likely level of ‘fertility failure’ and other losses, allowing them to better estimate replacement requirements. If the right number of replacements are reared to balance with longer productive life of cows in the herd then the overall percentage of first lactation cows in a herd should decrease. The percentage of first lactation cows is an available parameter in InterHerd+ and could be monitored as an indicator of overall herd efficiency. The average age or lactation number of cows on exit is also useful: an increase in these parameters would indicate increased longevity. However there are two factors that could be linked with the current decline in these parameters. Culling cows younger to make room for first-calved replacements will clearly reduce average exit age, but if cows are retained for more lactation and yet first-calved cows are over-supplied, a higher proportion of first lactation cow removals would also reduce, or at least hold static, the average exit age. If a high proportion of first lactation exits occur early in lactation, as found in this study, then the percentage of first lactation cows parameter will not accurately reflect the level of over-supply of replacements.

The deeper insights gained from the analyses presented in this paper demonstrate the potential value of extending the range and detail of parameters available routinely from milk recording data to include the percentages of cows progressing from first to second lactation, second to third, third to fourth and so on. Monitoring these parameters would allow a clearer understanding of replacement efficiency and longevity in the herd. Breaking exit data down according to the fertility stage of the cow on exit would add even more useful information. All of these more detailed parameters could feasibly be derived from currently available routine milk recording data. It would also improve information on exits if the type of exit was more fully recorded (as ‘end of life cull’, for ‘further production in another herd’ etc.) but this would require active input by the farmer in each case. Such monitoring of enforced and voluntary culls would allow better prediction of the number of replacement heifers required over the following few years to maintain the herd size and hence where to use dairy or beef semen on cows to avoid over production of replacements.

## Conclusions

Overall, 82.6% of first lactation cows progressed to second lactation within the same herd. Our data suggested that age at first calving and days post-partum at conception were the most important factors that may be considered by farmers when deciding which cows to retain, with 305 day yield and average SCC being of less importance. There is a need for more detailed monitoring of cows’ lifetime performance and progression from lactation to lactation in order to attain the most efficient longevity of dairy cows and reduce the inefficient over-supply of replacements. Future work could be expanded to later parities to better understand the individual cow factors affecting the balance of the decision to retain an older cow or replace it with an available first calved cow. More detailed exit information would allow better prediction of the number of replacement heifers required and avoid over production of replacements. Alongside this there should be ranking of cows according to their worth as breeders of replacements, based on their pedigree and performance in the herd.

## Supporting information

S1 Table Raw first lactation cow data used within this study. (PDF)

S2 Table The criteria and parity outcomes assigned for ALL the first lactation cattle calving in 2020. (PDF)

S3 Table Last recorded fertility status of the unknown outcomes. (XLSX)
