# Supplementary material for: Milk recording data indicates the importance of fertility, including age at first calving, on the progression of first lactation cows to second lactation
Source: PLoS One. 2024 Jan 29;19(1):e0297657. doi: 10.1371/journal.pone.0297657 (PMC10824451; doi:10.1371/journal.pone.0297657)
Supplement: S1 Table — (PDF) [file pone.0297657.s001.pdf]

S1 Table. The criteria and parity outcomes assigned for ALL the first lactation cattle calving in 2020.

Serve	Conc	Recal	Exit	Parity outcome	n	(% of total)	n Died
Yes	Yes	Yes	*	1. Re-calved	24,051	(79.2%)	0
Yes	Yes	No	Yes	2. Conceived: EXIT	1,419	(4.7%)	35
Yes	No	No	Yes	3. Served not conceived: EXIT	1,744	(5.7%)	32
No	No	No	Yes	4. not served: EXIT	1,914	(6.3%)	38
No	No	?	?	X1. Not served	594	(2.0%)	??
Yes	No	?	?	X2. Served not conceived	323	(1.1%)	??
Yes	Yes	?	?	X3. Conceived	315	(1.0%)	??
				Total	30,360	(100.0%)	105

** 14 re-calved cows were recorded as SOLD with an exit date the same as their re-calve date*

IH+ includes an updatable ‘fertility status’ that users can select and update from fixed options. The latest fertility status of the 1,232 cases lacking either a re-calve or exit date are shown in table S(ii).

381 (31%) were recorded as ‘RE-CALVED’ (but with no service dates or conception date recorded);

343 (28%) were recorded as ‘SOLD’ (of these, 108 had a recorded conception date);

161 (13%) were recorded as ‘PREGNANT’ with a conception date recorded;

135 (11%) were recorded as ‘BARREN’ or ‘PD NEG’;

174 (14%) were recorded as ‘SERVED’ (of these, 19 had a recorded conception date);

the remaining 38 (3%) had no fertility events recorded so remained with the default status ‘NH/NS’ (No Heat / No Service).
