# Supplementary material for: Milk recording data indicates the importance of fertility, including age at first calving, on the progression of first lactation cows to second lactation
Source: PLoS One. 2024 Jan 29;19(1):e0297657. doi: 10.1371/journal.pone.0297657 (PMC10824451; doi:10.1371/journal.pone.0297657)
Supplement: S2 Table — (PDF) [file pone.0297657.s002.pdf]

S2 Table. Last recorded fertility status of the unknown outcomes.

Category	Barren	NH/NS	PD Neg	Pregnant	Re-calved	Served	Sold	Overall
X1. Not served	20 (3.4%)	38 (6.4%)	0 (0.0%)	0 (0.0%)	381 (64.1%)	0 (0.0%)	155 (26.1%)	594 (100.0%)
X2. Served not conceived	84 (26.0%)	0 (0.0%)	4 (1.2%)	0 (0.0%)	0 (0.0%)	155 (48.0%)	80 (24.8%)	323 (100.0%)
X3. Conceived	27 (8.6%)	0 (0.0%)	0 (0.0%)	161 (51.1%)	0 (0.0%)	19 (6.0%)	108 (34.3%)	315 (100.0%)
Overall	131 (10.6%)	38 (3.1%)	4 (0.3%)	161 (13.1%)	381 (30.9%)	174 (14.1%)	343 (27.8%)	1,232 (100.0%)
